# Efficacy and Safety of Bisphosphonates for Low Bone Mineral Density After Kidney Transplantation: A Meta-Analysis

**DOI:** 10.1097/MD.0000000000002679

**Published:** 2016-02-08

**Authors:** Shun-Li Kan, Guang-Zhi Ning, Ling-Xiao Chen, Yong Zhou, Jing-Cheng Sun, Shi-Qing Feng

**Affiliations:** From the Department of Orthopaedics, Tianjin Medical University General Hospital, 154 Anshan Road, Heping District, Tianjin, China.

## Abstract

In patients with low bone mineral density (BMD) after kidney transplantation, the role of bisphosphonates remains unclear. We performed a systematic review and meta-analysis to investigate the efficacy and safety of bisphosphonates.

We retrieved trials from PubMed, EMBASE, and the Cochrane Central Register of Controlled Trials (CENTRAL) from inception through May 2015. Only randomized controlled trials that compared bisphosphonate-treated and control groups of patients with low bone mineral density after kidney transplantation were included. The primary outcomes were the percent change in BMD, the absolute change in BMD, and the BMD at the end of study at the lumbar spine. The results were expressed as the mean difference (MD) or relative risk (RR) with the 95% confidence interval (CI). We used a random-effects model to pool the outcomes.

We included 17 randomized controlled trials with 1067 patients. Only 1 included trial was found to be at low risk of bias. The rest of the included studies were found to have high to uncertain risk of bias. Compared with the control group, those who received bisphosphonates had a significant increase in percent change in BMD (mean difference [MD] = 5.51, 95% confidence interval [CI] 3.22–7.79, *P* < 0.00001) and absolute change in BMD (MD = 0.05, 95% CI 0.04–0.05, *P* < 0.00001), but a nonsignificant increase in BMD at the end of the study (MD = 0.02, 95% CI −0.01 to 0.05, *P* = 0.25) at the lumbar spine. Bisphosphonates resulted in a significant improvement in percent change in BMD (MD = 4.95, 95% CI 2.57–7.33, *P* < 0.0001), but a nonsignificant improvement in absolute change in BMD (MD = 0.03, 95% CI −0.00 to 0.06, *P* = 0.07) and BMD at the end of the study (MD = −0.01, 95% CI −0.04 to 0.02, *P* = 0.40) at the femoral neck. No significant differences were found in vertebral fractures, nonvertebral fractures, adverse events, and gastrointestinal adverse events.

Bisphosphonates appear to have a beneficial effect on BMD at the lumbar spine and do not significantly decrease fracture events in recipients. However, the results should be interpreted cautiously due to the lack of robustness and the heterogeneity among studies.

## Abstract 2

Supplemental Digital Content is available in the text

## INTRODUCTION

Kidney transplantation is an optimal choice for patients suffering end-stage renal disease.^1^ It was estimated that >16,000 patients underwent kidney transplants throughout America in 2012.^2^ Considering the increasingly long duration of survival,^3^ low bone mineral density (BMD) after kidney transplantation has been paid increasing attention and is most commonly declared during the first year after transplantation. Torregrosa and colleagues^4^ reported that BMD decreased by 7% to 10% during the first year after kidney transplantation. As a result, the incidence of fractures has increased.^5,6^ Previous studies have reported a fracture incidence of 5% to 60% ^7,8^ after successful renal transplantation. Thus, the prevention and treatment of these complications have been prioritized in postoperative care.

The reasons for low BMD are multifaceted, and immunosuppressive agents may be major contributors.^9^ Additionally, persistent hyperparathyroidism and calcineurin inhibitors may result in the rapid loss of bone mass.^10^ Bisphosphonates, by decreasing the number of osteoclasts and inhibiting their activity, are effective in the treatment of glucocorticoid-induced osteoporosis.^11^ However, in patients with low BMD after kidney transplantation, the role of bisphosphonates remains unclear. Two previous meta-analyses^12,13^ included nonrandomized trials, which compromised the credibility of the results. Some recently published randomized controlled trials on the topic have conveyed conflicting results.^14,15^ Therefore, we performed a systematic review and meta-analysis of randomized controlled trials to investigate the efficacy of bisphosphonates for low bone mineral density after kidney transplantation.

## MATERIALS AND METHODS

### Search Strategy and Study Selection

We retrieved studies reporting randomized controlled trials of bisphosphonates for low bone mineral density after kidney transplantation from PubMed, EMBASE, and the Cochrane Central Register of Controlled Trials (CENTRAL). The retrievals were last updated on May 19, 2015. The medical subject heading and text words used were “renal transplantation” or “kidney transplantation,” “bisphosphonates” or the generic names of multifarious bisphosphonates. These terms were connected by means of the Boolean operators “AND” and “OR.” The detailed literature search was shown in Supplemental Table 1. There were no restrictions with respect to sex, language, geography, and follow-up time. Reference lists of valuable trials, reviews, meta-analyses, and reports were also manually searched for additional relevant studies. All titles and abstracts of the initially identified studies were assessed for eligibility. Two reviewers (KSLK and NGZN) independently assessed the full text of potentially eligible articles based on the inclusion criteria. Disagreements were resolved by referring to a third reviewer (SQF).

### Eligibility Criteria

(1) Participants: only studies enrolling participants receiving cadaveric or living renal allografts were included. Studies of recipients receiving any transplantation other than a renal transplantation, including studies of kidney-pancreas transplantations, were excluded. Studies enrolling participants over the age of 18 were included. Studies with any immunosuppression regimen after kidney transplantation were included.

(2) Interventions: the intervention in the experimental group was the oral, intramuscular, or intravenous administration of bisphosphonates, alone or in association with calcium and/or vitamin D.

(3) Comparisons: the intervention in the control group was placebo or no treatment, alone, or in association with calcium and/or vitamin D.

(4) Outcomes: the percent change in BMD, the absolute change in BMD, and the BMD at the end of the study at the lumbar spine and femoral neck, vertebral fractures, nonvertebral fractures, adverse events, and gastrointestinal adverse events were collected as the outcomes.

(5) Study design: only randomized controlled trials were included in our study. The more recent or most complete records were included if there were multiple publications for the same study.

### Data Extraction and Outcome Measures

For each study, data extraction was performed independently by 2 investigators (SLK and GZN). Data regarding the study author, year of publication, intervention and comparison, follow-up, patient characteristics, sample size, and outcome, were collected. Intervention details including the intervention method, intervention initiation timing, intervention exposure time and intervention dose, were also recorded. The corresponding authors or the first authors were contacted for additional information. Consensus between the 2 assessors (SLK and GZN) was used to resolve any discrepancy.

The primary outcomes of interest in the meta-analysis were the percent change in BMD, the absolute change in BMD, and the BMD at the end of the study at the lumbar spine after successful renal transplantation because these were the most commonly used primary outcomes in studies appraising the effect of bisphosphonates in treating or preventing low BMD. When the percent change in BMD, the absolute change in BMD, and the BMD at the end of the study were reported at different follow-up intervals, we used data from the longest complete follow-up. Secondary outcomes included the percent change in BMD, the absolute change in BMD, and the BMD at the end of the study at the femoral neck, vertebral fractures, nonvertebral fractures, adverse events, and gastrointestinal adverse events. The fracture events identified by radiographs were accepted as the evidence of vertebral and nonvertebral fractures. The result obtained from 1 of the 3 methods measuring BMD that included the most studies was the final result. If there were 2 or 3 ways including the same studies, we regarded the conservative result as the final result.

Perprotocol data were used in the analysis of the percent change in BMD, the absolute change in BMD, and the BMD at the end of the study whenever possible. Intention-to-treat data were used in the other variables. If the means, standard deviations (SDs), or standard error of the means (SEMs) were not available in the text of articles, we extracted data from the diagrams and tables, if available.^16^

### Risk of Bias Assessment

The risk of bias tool was performed to assess the risk of bias of individual studies in accordance with the Cochrane Handbook for Systematic Reviews of Interventions (version 5.1.0).^16^ Two reviewers (SLK and LXC) independently reviewed all studies. The domains of assessment for the outcomes were sequence generation (selection bias), allocation sequence concealment (selection bias), the blinding of participants and personnel (performance bias), the blinding of outcome assessment (detection bias), incomplete outcome data (attrition bias), selective outcome reporting (reporting bias), and other biases (baseline balance and fund). Each of the domains was judged as a low risk of bias, a high risk of bias, or an unclear risk of bias. A trial was regarded as having a high risk of bias if 1 or more key domains were considered to be at high risk. A trial was regarded as having a low risk of bias if all key domains were considered to be at low risk. Otherwise, they were regarded as having an unclear risk of bias.^17^

### Quality of Evidence Assessment

The Grading of Recommendations, Assessment, Development and Evaluation (GRADE) methodology^18^ was used to assess the quality of evidence. GRADE Working Group grades of evidence were classified as high, moderate, low, or very low based on the judgment for the outcome with respect to the risk of bias and to inconsistency, indirectness, imprecision, and publication bias.^18,19^ Two investigators (SLK and LXC) independently assessed the quality of evidence, and any disagreements were solved by discussion and consensus or by referring to a third reviewer (SQF). Summary tables were constructed using GRADE Pro, version 3.6.

### Statistical Analysis

For the percent change in BMD, the absolute change in BMD, and the BMD at the end of the study, we calculated the mean difference (MD) and 95% confidence interval (CI). The relative risk (RR) and corresponding 95% CI were used for vertebral fractures, nonvertebral fractures, adverse events, and gastrointestinal adverse events. A random-effects model was used to pool data for summary estimates.^20^ Heterogeneity across trials was evaluated using the *I*^2^ statistic^21^ and chi-square test.^22^ Heterogeneity was considered significant if *I*^2^ >50%. For alterations of the BMD at the lumbar spine, subgroup analyses were implemented according to the administration method (intravenous versus peroral), study duration (short term [<12 months] vs long term [≥12 months]), bisphosphonates usage (continuous or intermittent), treatment indication (prevention versus treatment of bone loss). Furthermore, we performed a metaregression analysis to evaluate the effect of study duration on alterations of the BMD at the lumbar spine when >10 trials were available.^23^ There were 3 methods for evaluating BMD changes, but subgroup and metaregression analyses were only performed for the method that was used in the most studies. To test publication bias, Egger's linear regression test was implemented for changes in BMD, and funnel plots were created to visualize possible asymmetry when the number of studies was >10.^24^*P* < 0.05 was considered statistically significant. All statistical analyses were performed using Review Manager, version5.3 (The Nordic Cochrane Centre, The Cochrane Collaboration, Copenhagen, 2014) and Stata, version 12.0 (Stata Corp, College Station, TX).

### Ethical Statement

As all analyses were grounded on previously published studies, ethical approval was not necessary.

## RESULTS

### Search for Studies

**FIGURE 1:**
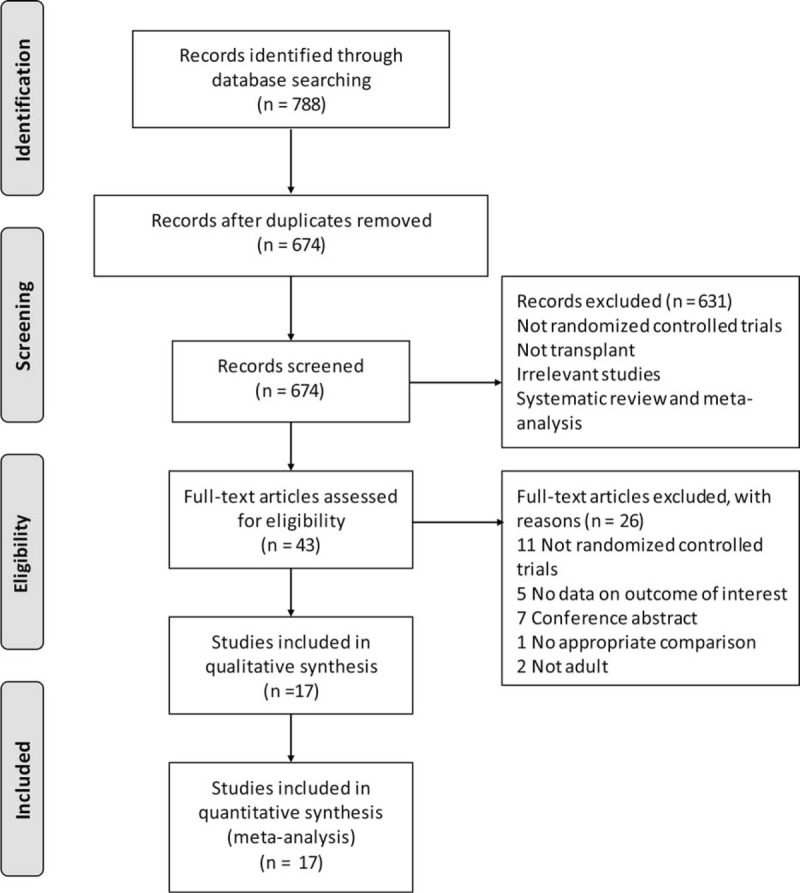
The flowchart of study selection.

The literature screening strategy is demonstrated in the flowchart (Figure 1). Initially, 788 relevant trials were identified; 114 were excluded due to duplicate reportage, and 631 were excluded at the title and abstract level. The full texts of 43 potentially eligible articles were evaluated based on the inclusion criteria. After assessing the full texts, 26 studies were excluded. Finally, a total of 17 eligible records ^14,15,25–41^ were included. Two identified publications provided data from the same trial.^35,42^

### Study Characteristics

There were 17 articles ^14,15,27–41^ included in our meta-analysis. All the articles were published in English between October 1998 and January 2012. The number of participants in the bisphosphonates and control groups was in the range of 8 to 66 (total = 521) and 8 to 63 (total = 546), respectively, with a total of 1067 participants. Our study identified 5 studies that used the bisphosphonate pamidronate,^15,29,34,35,40^ 5 trials that used alendronate,^14,32,33,37,38^ 3 trials that used risedronate,^27,30,31^ 2 trials that used ibandronate,^28,39^ 1 trial that used zoledronate,^36^ and 1 trial that used clodronate.^41^ Other than Giannini,^38^ Jeffery,^33^ Koc,^37^ Trabulus,^14^ and Grotz,^41^ who applied daily alendronate and clodronate, the other investigators administered bisphosphonates in a cyclic fashion. Moreover, the participants included in 15^14,15,28–34,36–41^ of 17 studies received daily calcium, and the participants included in^14,15,27–34,37,38,40^ of 17 studies received vitamin D or its analogs. Patients included in 15^14,15,27–31,33–39,41^ of 17 studies received immunosuppressive therapy comprising glucocorticoids, cyclosporine, or tacrolimus with or without mycophenolate mofetil or azathioprine, and 2 studies^32,40^ did not report the use of immunosuppressive therapy. There was no significant difference between the study and control groups in immunosuppressive therapy. Table 1 outlined the baseline characteristics of the included studies.

**TABLE 1:**
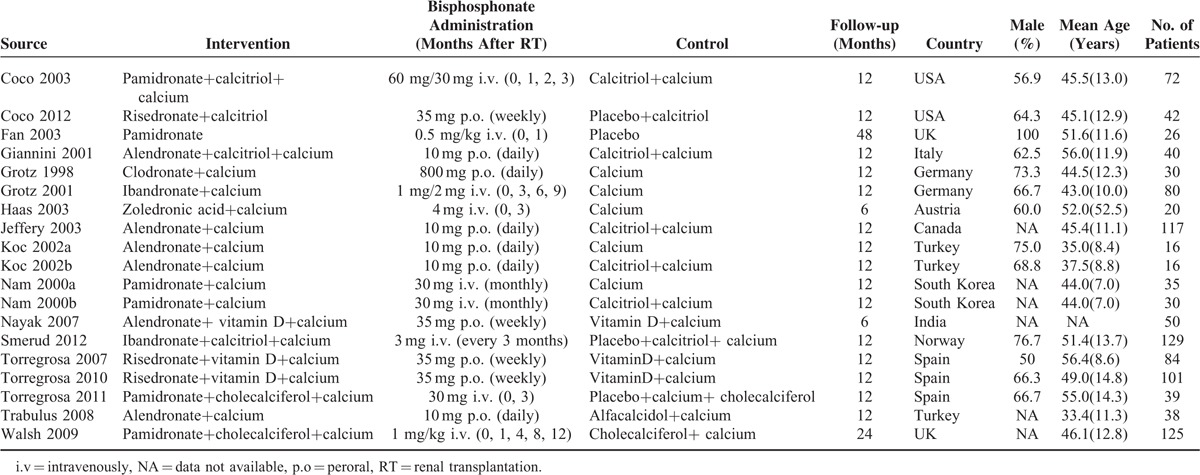
Baseline Characteristics of Studies Included in the Meta-Analysis

### Risk of Bias in the Included Studies

**FIGURE 2:**
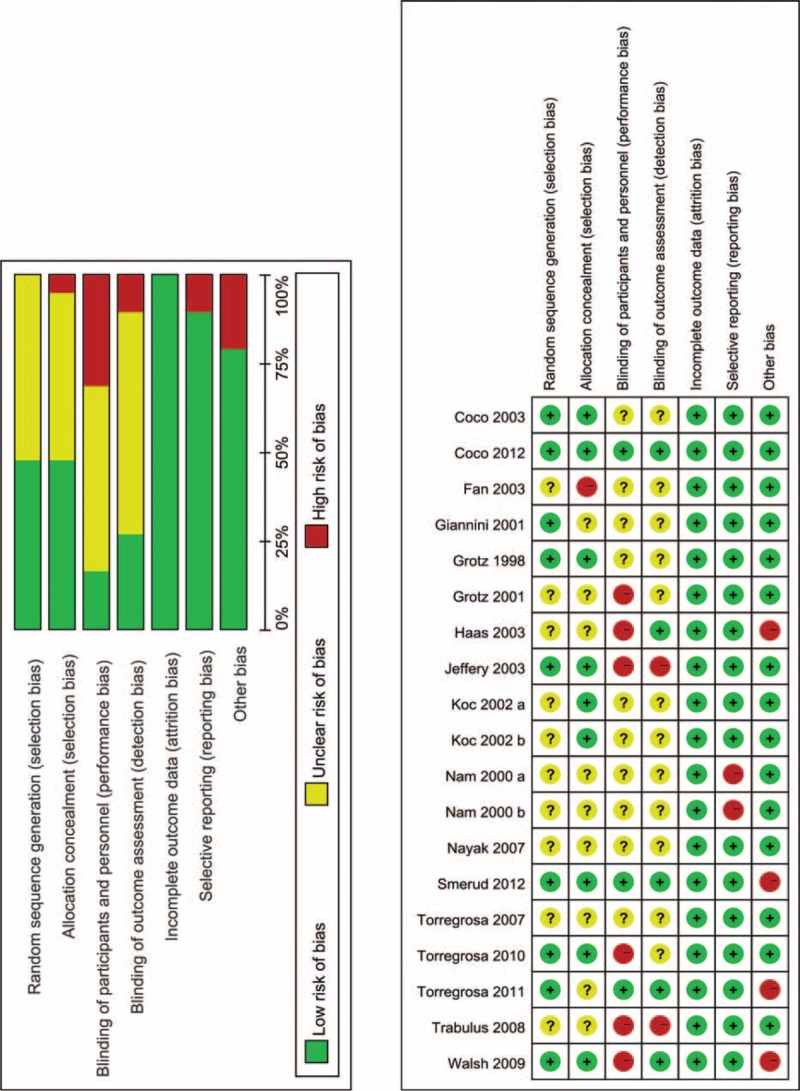
Risk of bias assessment of each included study.

The risk of bias assessment of included studies, together with potential sources of bias, was outlined in Figure 2. Randomized sequence generation was conducted adequately in 9 studies.^15,27–30,33,34,38,41^ Allocation concealment was conducted adequately in 8 studies.^15,27,28,30,33,34,37,41^ Four studies^15,28,29,36^ received a grant from industry or another type of profit support. Only 1 included trial ^27^ was found to have a low risk of bias. The remaining included studies were found to have a high or an uncertain risk of bias.

### Quality of Evidence Assessment

Supplemental Table 2, includes a summary of the findings, including outcomes together with an appraisal of the quality of evidence according to the GRADE approach. The GRADE level of evidence was low for the percent change in BMD at the lumbar spine and femoral neck, for vertebral fractures and for adverse events and was moderate for the absolute change in BMD at the lumbar spine and femoral neck, for the BMD at the end of the study at the lumbar spine and femoral neck, for nonvertebral fractures, and for gastrointestinal adverse events.

### Percent Change in BMD at the Lumbar Spine and Femoral Neck

**FIGURE 3:**
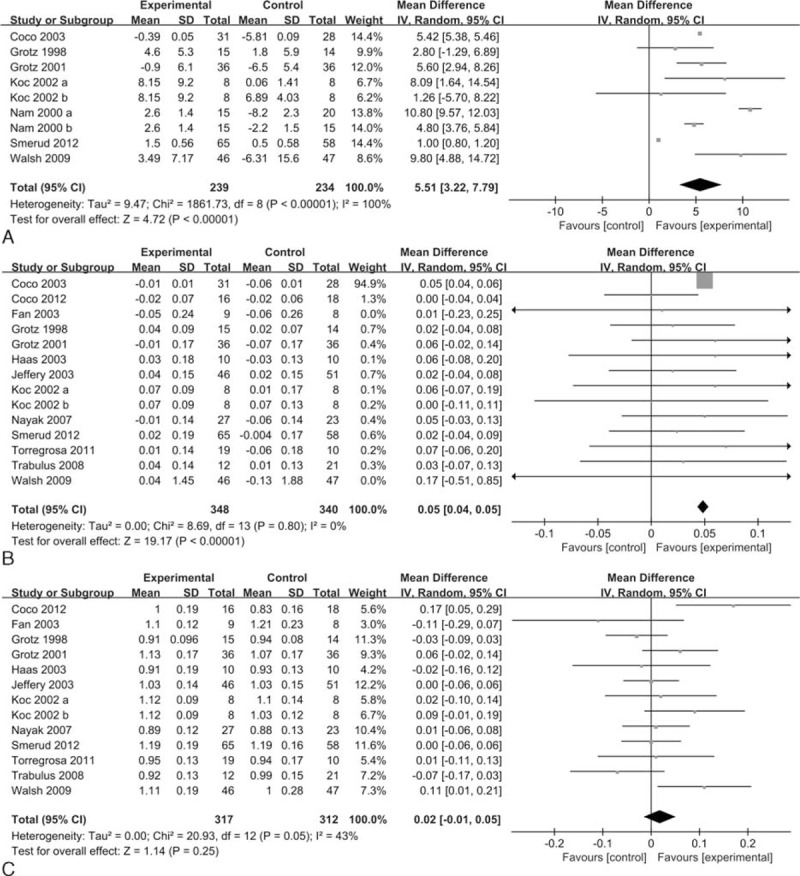
Forest plots of the included studies comparing percent change in BMD (A), absolute change in BMD (B), and BMD at the end of the study (C) at the lumbar spine in patients who received bisphosphonates and those who did not. BMD = bone mineral density.

Seven studies with 9 groups including 533 patients were used to compute the pooled estimate to evaluate the percent change in BMD at the lumbar spine. Compared with the control, bisphosphonates were associated with a significant increase in the percent change in BMD at the lumbar spine (MD = 5.51, 95% CI 3.22–7.79, *P* < 0.00001; *I*^2^ = 100%) (Figure 3A).

**FIGURE 4:**
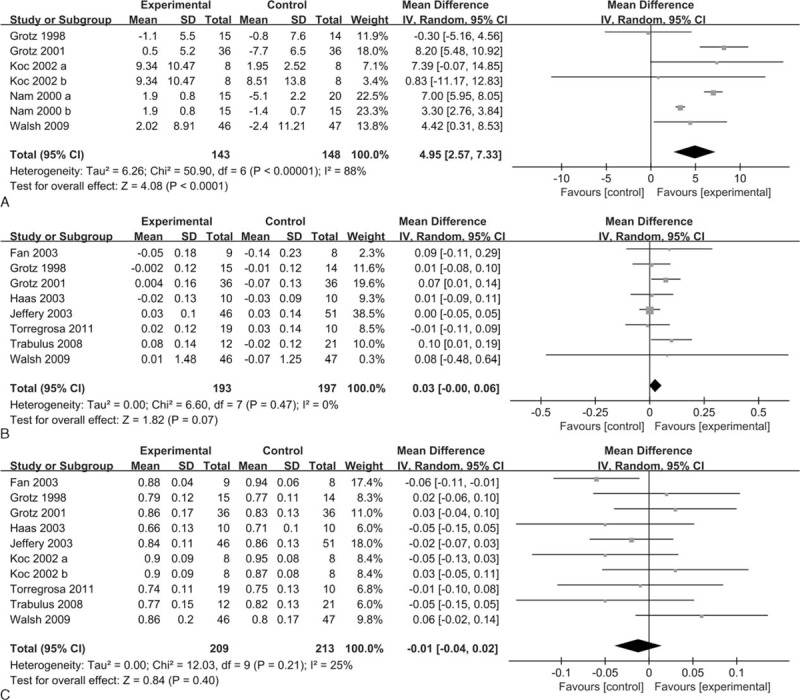
Forest plots of the included studies comparing percent change in BMD (A), absolute change in BMD (B), and BMD at the end of the study (C) at the femoral neck in patients who received bisphosphonates and those who did not. BMD = bone mineral density.

Five studies (n = 332) with 7 groups were included in the analysis of the percent change in BMD at the femoral neck. The percent change in BMD at the femoral neck was significantly increased with bisphosphonates compared with the control (MD = 4.95, 95% CI 2.57–7.33, *P* < 0.0001; *I*^2^ = 88%) (Figure 4A).

### Absolute Change in BMD at the Lumbar Spine and Femoral Neck

Data on the absolute change in BMD at the lumbar spine were available from 13 studies (n = 800) with 14 groups. Bisphosphonates were associated with an increased absolute change in BMD at the lumbar spine (MD = 0.05, 95% CI 0.04–0.05, *P* < 0.00001; *I*^2^ = 0%) (Figure 3B). The funnel plot was visually inspected and did expose some asymmetry (Supplemental Figure 1), and the Egger test did not show a significant trend toward publication bias among the included studies (*P* = 0.09).

Bisphosphonates did not result in a significant improvement in the absolute change in BMD at the femoral neck across 8 trials including a total of 475 patients (MD = 0.03, 95% CI −0.00 to 0.06, *P* = 0.07; *I*^2^ = 0%) (Figure 4B).

### BMD at the End of the Study at the Lumbar Spine and Femoral Neck

Data on the BMD at the end of the study at the lumbar spine were available in 12 studies (n = 728) with 13 groups. No significant difference was found in the BMD at the end of the study at the lumbar spine between bisphosphonates and control (MD = 0.02, 95% CI −0.01 to 0.05, *P* = 0.25; *I*^2^ = 43%) (Figure 3C). The funnel plot was visually inspected and did expose some asymmetry (Supplemental Figure 2), and no statistical evidence of publication bias was found among the included studies, as evaluated by the Egger test (*P* = 0.50).

Nine studies with 10 groups including 507 patients provided data for the BMD at the end of the study at the femoral neck. Compared with the control, bisphosphonates were not associated with a significant increase in the BMD at the end of the study at the femoral neck (MD = −0.01, 95% CI −0.04 to 0.02, *P* = 0.40; *I*^2^ = 25%) (Figure 4C).

### Vertebral Fractures

**FIGURE 5:**
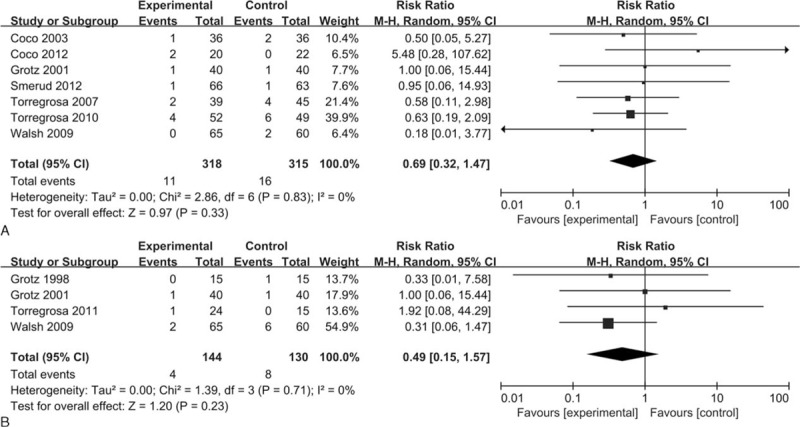
Forest plots of the included studies comparing vertebral fractures (A) and nonvertebral fractures (B) in patients who received bisphosphonates and those who did not.

A total of 7 studies including 633 patients that evaluated vertebral fractures following bisphosphonates did not indicate a significant decrease in the incidence of vertebral fractures (RR = 0.69, 95% CI 0.32–1.47, *P* = 0.33; *I*^2^ = 0%) (Figure 5A).

### Nonvertebral Fractures

Bisphosphonates did not reduce the incidence of nonvertebral fractures across 4 trials that reported nonvertebral fractures from 274 patients (RR = 0.49, 95% CI 0.15–1.57, *P* = 0.23; *I*^2^ = 0%) (Figure 5B).

### Adverse Events

**FIGURE 6:**
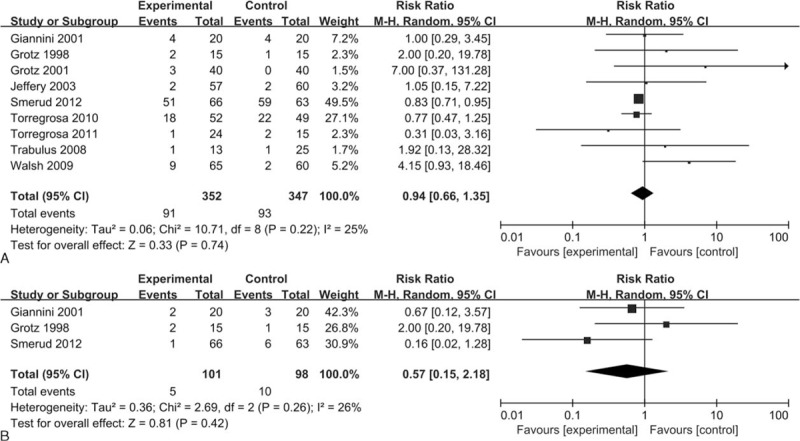
Forest plots of the included studies comparing adverse events (A) and gastrointestinal adverse events (B) in patients who received bisphosphonates and those who did not.

Nine studies (n = 699) reported on adverse events. We found no significant differences in the incidence of adverse events between bisphosphonates and control (RR = 0.94, 95% CI 0.66–1.35, *P* = 0.74; *I*^2^ = 25%) (Figure 6A). Similarly, no significant differences were found in the risk of gastrointestinal adverse events between bisphosphonates and control across 3 studies including 199 patients (RR = 0.57, 95% CI 0.15–2.18, *P* = 0.42; *I*^2^ = 26%) (Figure 6B).

### Subgroup and Metaregression Analyses

As the absolute change in BMD was included in more studies than was the percent change in BMD at the lumbar spine, subgroup and metaregression analyses were performed for the absolute change in BMD at the lumbar spine. Subgroup analyses demonstrated that bisphosphonates were significantly more effective than the control in the intravenous treatment groups, long-term treatment groups and intermittent treatment groups as well as in preventing osteopenia/osteoporosis (Supplemental Figures 3–6). Bisphosphonates did not show superiority over the control in certain subgroups, including the peroral treatment groups, short-term treatment groups, and continuous treatment groups, or in treating bone loss (Supplemental Figures 3–6).

Metaregression demonstrated no effect of study duration in improving lumbar spine BMD (Supplemental Figure 7).

## DISCUSSION

In this meta-analysis, we assessed the efficacy and safety of bisphosphonates for low BMD after successful renal transplantation. By incorporating the most recent evidence from randomized controlled trials, we constructed the largest database regarding the use of bisphosphonates in the prevention and treatment of low BMD in patients undergoing renal transplantation.

In our meta-analysis of 17 randomized controlled trials, patients who received bisphosphonates after renal transplantation experienced a significant improvement in the absolute change in BMD at the lumbar spine; however, they did not experience a significant increase of BMD at the end of the study at the femoral neck. The use of bisphosphonates was not associated with a decreased risk of vertebral and nonvertebral fractures and an increase of adverse events and gastrointestinal adverse events.

Our findings demonstrated that bisphosphonates could improve BMD but could not reduce the fracture incidence after kidney transplantation. In a controlled clinical trial, Grotz and colleagues^5^ found that the BMD of kidney transplant recipients did not correlate directly with the fracture risk. Thus, whether the positive effect of bisphosphonates on BMD results in reduced fracture morbidity could not be concluded. In addition, Palmer and colleagues^13^ determined that the integration of any intervention for low BMD after kidney transplantation showed a modest decrease in the relative risk of fracture by 49% following 6 to 12 months of treatment. This finding was dependent on the inclusion of a large enough number of participants in the combined analysis, which allowed adequate power to determine a significant change in the fracture rate between any intervention and placebo/no treatment. Findings by Naylor et al^43,44^ demonstrated that kidney transplant recipients may have a lower fracture risk than previously suggested in the literature. This opinion was also supported by other studies.^45,46^ Thus, further adequately powered studies, with high quality, adequate sample sizes and selecting fracture events as the primary outcome, are required to determine the impact of bisphosphonates on risk of fracture.

In a previous meta-analysis of 5 studies comparing bisphosphonates and control in patients after renal transplantation, Mitterbauer and colleagues^12^ found that bisphosphonates appeared to be efficacious in preventing bone loss without major side effects in the early period after renal transplantation. However, 1 of the included studies conducted by Kovac and colleagues ^47^ was a letter to the editor. In this study, there was not enough information regarding randomized controlled trials, which may harm the reliability of the outcomes. Furthermore, all 5 studies had modest sample sizes (n < 100), which may have resulted in overestimation of the treatment effect compared with larger samples. Therefore, their outcomes may not be regarded as definitive. The current meta-analysis demonstrated that bisphosphonates appeared to reduce bone loss, which confirmed the results of Mitterbauer and colleagues.^12^

To better investigate the effect of bisphosphonates in patients after kidney transplantation, Palmer and colleagues^13^ performed a Cochrane systematic review and meta-analysis to assess the use of interventions for treating bone disease following renal transplantation. This review included 12 randomized controlled studies, including 9 studies described here^33–41^ and demonstrated that bisphosphonates may protect against immunosuppression-induced reductions in BMD at the lumbar spine and femoral neck and prevent fracture. The current meta-analysis did not include 4 studies enrolled in the previous analysis because 1 was an abstract rather than a full text,^48^ 1 was not described as randomized,^4^ and 1 mainly included children and adolescents,^49^ which may harm the reliability of the results. The results of the current meta-analysis indicated that bisphosphonates had a favorable effect on the BMD at the lumbar spine. The results of our meta-analysis generally concur and further strengthen the previous findings by including another 8 recently published studies.^14,15,27–32^

Notably, differences exist between our meta-analysis and the previous ones. The 2 previous meta-analyses included studies that may be not randomized trials, which are thus subject to bias. In addition, the data from nonrandomized and randomized trials were pooled together. To offer more reliable evidence and minimize potential bias, we included only randomized trials with full text and that focused on adult patient population. Our meta-analysis of 17 randomized trials including 1067 participants demonstrated that bisphosphonates were beneficial to BMD at the lumbar spine rather than at the femoral neck. No significant differences were found between the bisphosphonates and control groups in vertebral fractures, nonvertebral fractures, adverse events, and gastrointestinal adverse events.

There are several potential limitations in this meta-analysis that should be taken into account. First, our analysis is based on 17 randomized controlled trials, but most of these trials have a modest sample size (n < 100). Compared with large sample size trials, small sample size trials are more likely to overestimate the treatment effect,^50^ which restricts the power of the inferences. Second, most of the included studies are not blinded or unclear so that only 1 included trial had a low risk of bias and the remaining ones were at high or uncertain risk of bias, which may generate bias and impact the effect sizes. Third, the characteristics of participants, the baseline data regarding BMD, and the bisphosphonates regimen (dosage, species, route, timing, and duration of administration) differ among the included studies. These factors may have a potential impact on the calculation of the percent change in BMD, the absolute change in BMD, and the BMD at the end of the study. Finally, some patients had diabetes mellitus, and the condition of diabetes mellitus had an impact on BMD.^6^ However, we could not abstract the data of these patients to conduct subgroup analysis. Although the number of patients with diabetes mellitus was small (4.5%), this may have influenced our results.

## CONCLUSIONS

Based on the current limited evidence, bisphosphonates appear to be effective for low BMD after kidney transplantation at the lumbar spine rather than that at the femoral neck. Furthermore, the use of bisphosphonates is not associated with significant changes in vertebral fractures, nonvertebral fractures, adverse events, and gastrointestinal adverse events. However, our results should be interpreted cautiously because the data were limited by the insufficient sample sizes of the studies and heterogeneity that existed among the studies. As our results are not robust, further larger randomized controlled trials are still needed to verify the efficacy of bisphosphonates for low BMD after kidney transplantation.

## Supplementary Material

Supplemental Digital Content
