# Supplementary material for: Efficacy and Safety of Bisphosphonates for Low Bone Mineral Density After Kidney Transplantation: A Meta-Analysis
Source: Medicine (Baltimore). 2016 Feb 8;95(5):e2679. doi: 10.1097/MD.0000000000002679 (PMC4748922; doi:10.1097/MD.0000000000002679)
Supplement: Supplemental Digital Content [file medi-95-e2679-s001.doc]

**Supplemental Table 1 Search Strategy**

**Source:** PubMed

**Searched on:** May 19, 2015

| **Search** | **Query** |
| --- | --- |
| #1 | "Diphosphonates"[Mesh] |
| #2 | (((((((((((((((((((((Tiludron*[Title/Abstract]) OR Olpadron*[Title/Abstract]) OR Medron*[Title/Abstract]) OR Incadron*[Title/Abstract]) OR clodron*[Title/Abstract]) OR aredia[Title/Abstract]) OR pamidron*[Title/Abstract]) OR reclast[Title/Abstract]) OR aclasta[Title/Abstract]) OR zomera[Title/Abstract]) OR zometa[Title/Abstract]) OR boniva[Title/Abstract]) OR zolendron*[Title/Abstract]) OR actonel[Title/Abstract]) OR ibandron*[Title/Abstract]) OR risedron*[Title/Abstract]) OR didronel[Title/Abstract]) OR etidron*[Title/Abstract]) OR fosamax[Title/Abstract]) OR alendron*[Title/Abstract]) OR diphosphonat*[Title/Abstract]) OR biphosphonat*[Title/Abstract] |
| #3 | #1 OR #2 |
| #4 | "Kidney Transplantation"[Mesh] |
| #5 | (((renal graft*[Title/Abstract]) OR kidney graft*[Title/Abstract]) OR renal transplant*[Title/Abstract]) OR kidney transplant*[Title/Abstract] |
| #6 | #4 OR #5 |
| #7 | (("Randomized Controlled Trial"[Publication Type]) OR "Randomized Controlled Trials as Topic"[Mesh]) OR random* |
| #8 | #3 AND #6 AND #7 |

Search strategy used for PubMed, adapted for Embase and the Cochrane Central Register of Controlled Trials.

**Supplemental Table 2 GRADE evidence profile**

| **Quality assessment** | | | | | | | **No of patients** | | **Effect** | | **Quality*** | **Importance** |
| --- | --- | --- | --- | --- | --- | --- | --- | --- | --- | --- | --- | --- |
|  |  |  |  |  |  |  |  |  |  |  |  |  |
| **No of studies** | **Design** | **Risk of bias** | **Inconsistency** | **Indirectness** | **Imprecision** | **Other considerations** | **Bisphosphonates** | **Control** | **Relative (95% CI)** | **Absolute** |  |  |
| **Percent change in BMD at the lumbar spine (follow-up 12-24 months; Better indicated by lower values)** | | | | | | | | | | | | |
| 7 | randomised trials | serious^1^ | serious^2^ | no serious indirectness | no serious imprecision | none | 239 | 234 | - | MD 5.51 higher (3.22 to 7.79 higher) | ⊕⊕OO LOW | CRITICAL |
| **Percent change in BMD at the femoral neck (follow-up 12-24 months; Better indicated by lower values)** | | | | | | | | | | | | |
| 5 | randomised trials | serious^1^ | serious^3^ | no serious indirectness | no serious imprecision | none | 143 | 148 | - | MD 4.95 higher (2.57 to 7.33 higher) | ⊕⊕OO LOW | CRITICAL |
| **Absolute change in BMD at the lumbar spine (follow-up 6-48 months; Better indicated by lower values)** | | | | | | | | | | | | |
| 13 | randomised trials | serious^1^ | no serious inconsistency | no serious indirectness | no serious imprecision | none | 348 | 340 | - | MD 0.05 higher (0.04 to 0.05 higher) | ⊕⊕⊕O MODERATE | CRITICAL |
| **Absolute change in BMD at the femoral neck (follow-up 6-48 months; Better indicated by lower values)** | | | | | | | | | | | | |
| 8 | randomised trials | serious^1^ | no serious inconsistency | no serious indirectness | no serious imprecision | none | 193 | 197 | - | MD 0.03 higher (0 to 0.06 higher) | ⊕⊕⊕O MODERATE | CRITICAL |
| **BMD at the end of the study at the lumbar spine (follow-up 6-48 months; Better indicated by lower values)** | | | | | | | | | | | | |
| 12 | randomised trials | serious^1^ | no serious inconsistency | no serious indirectness | no serious imprecision | none | 317 | 312 | - | MD 0.02 higher (0.01 lower to 0.05 higher) | ⊕⊕⊕O MODERATE | CRITICAL |
| **BMD at the end of the study at the femoral neck (follow-up 6-48 months; Better indicated by lower values)** | | | | | | | | | | | | |
| 9 | randomised trials | serious^1^ | no serious inconsistency | no serious indirectness | no serious imprecision | none | 209 | 213 | - | MD 0.01 lower (0.04 lower to 0.02 higher) | ⊕⊕⊕O MODERATE | CRITICAL |
| **Vertebral fractures (follow-up 12-24 months)** | | | | | | | | | | | | |
| 7 | randomised trials | serious^1^ | no serious inconsistency | no serious indirectness | serious^4^ | none | 11/318  (3.5%) | 16/315  (5.1%) | RR 0.69 (0.32 to 1.47) | 16 fewer per 1000 (from 35 fewer to 24 more) | ⊕⊕OO LOW | CRITICAL |
|  |  |  |  |  |  |  |  | 3.3% |  | 10 fewer per 1000 (from 22 fewer to 16 more) |  |  |
| **Non-vertebral fractures (follow-up 12-24 months)** | | | | | | | | | | | | |
| 4 | randomised trials | serious^1^ | no serious inconsistency | no serious indirectness | no serious imprecision | none | 4/144  (2.8%) | 8/130  (6.2%) | RR 0.49 (0.15 to 1.57) | 31 fewer per 1000 (from 52 fewer to 35 more) | ⊕⊕⊕O MODERATE | CRITICAL |
|  |  |  |  |  |  |  |  | 4.6% |  | 23 fewer per 1000 (from 39 fewer to 26 more) |  |  |
| **Adverse events (follow-up 12-24 months)** | | | | | | | | | | | | |
| 9 | randomised trials | serious^1^ | no serious inconsistency | no serious indirectness | serious^5^ | none | 91/352  (25.9%) | 93/347  (26.8%) | RR 0.94 (0.66 to 1.35) | 16 fewer per 1000 (from 91 fewer to 94 more) | ⊕⊕OO LOW | IMPORTANT |
|  |  |  |  |  |  |  |  | 6.7% |  | 4 fewer per 1000 (from 23 fewer to 23 more) |  |  |
| **Gastrointestinal adverse events (follow-up 12 months)** | | | | | | | | | | | | |
| 3 | randomised trials | serious^1^ | no serious inconsistency | no serious indirectness | no serious imprecision | none | 5/101  (5%) | 10/98  (10.2%) | RR 0.57 (0.15 to 2.18) | 44 fewer per 1000 (from 87 fewer to 120 more) | ⊕⊕⊕O MODERATE | IMPORTANT |
|  |  |  |  |  |  |  |  | 9.5% |  | 41 fewer per 1000 (from 81 fewer to 112 more) |  |  |

Note: BMD=bone mineral density, CI=confidence interval, MD=mean difference.

^1^ Most trials were judged to be at high or unclear risk of bias.
^2^ Significant heterogeneity (I^2^ = 100%) was found.
^3^ Significant heterogeneity (I^2^ = 88%) was found.
^4^ RR with 95% CI for one trial was 5.48 (0.28-107.62).
^5^ RR with 95% CI for one trial was 7.00 (0.37-131.28).

*GRADE Working Group grades of evidence: high quality = further research is very unlikely to change our confidence in the estimate of effect; moderate quality = further research is likely to have an important impact on our confidence in the estimate of effect and may change the estimate; low quality = further research is very likely to have an important impact on our confidence in the estimate of effect and is likely to change the estimate; very low quality = we are very uncertain about the estimate.

Supplemental Figure 1 Funnel plot of absolute change in BMD at lumbar spine of the included studies comparing bisphosphonates with control


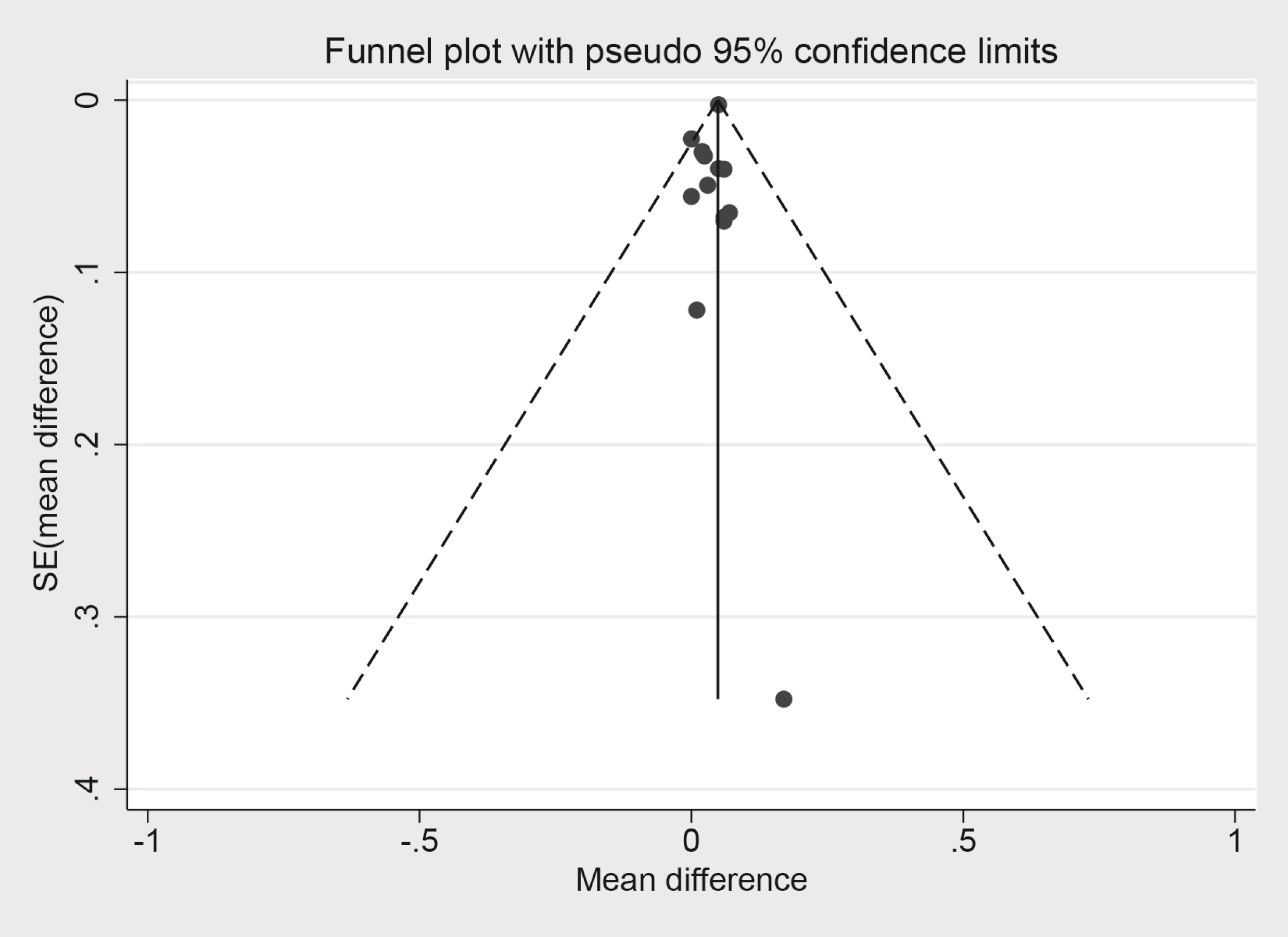


Supplemental Figure 2 Funnel plot of BMD at end of study at lumbar spine of the included studies comparing bisphosphonates with control


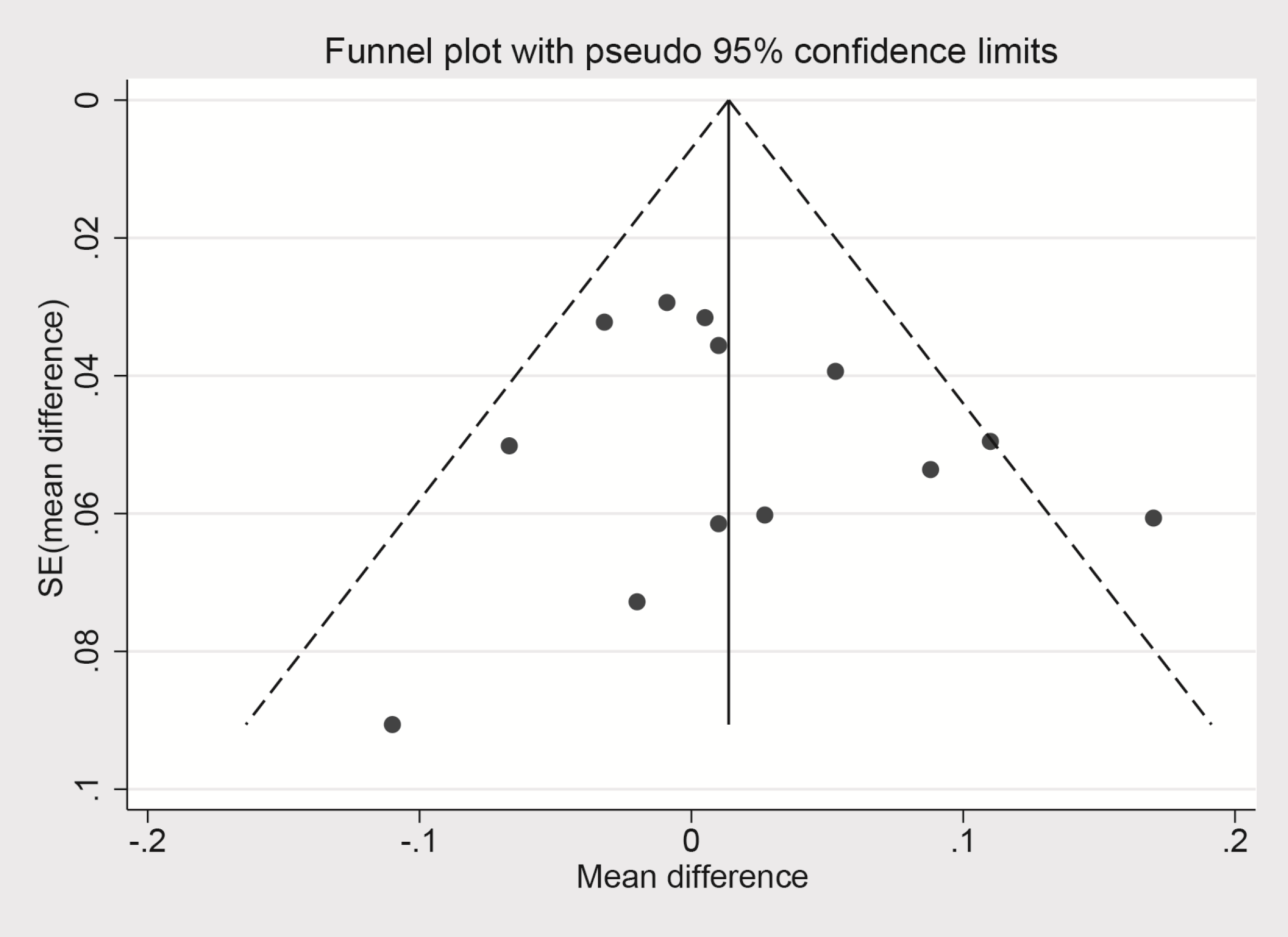


Supplemental Figure 3 Forest plot of absolute change in BMD at lumbar spine by subgroup analysis of administration method (intravenous versus peroral): bisphosphonates vs. control


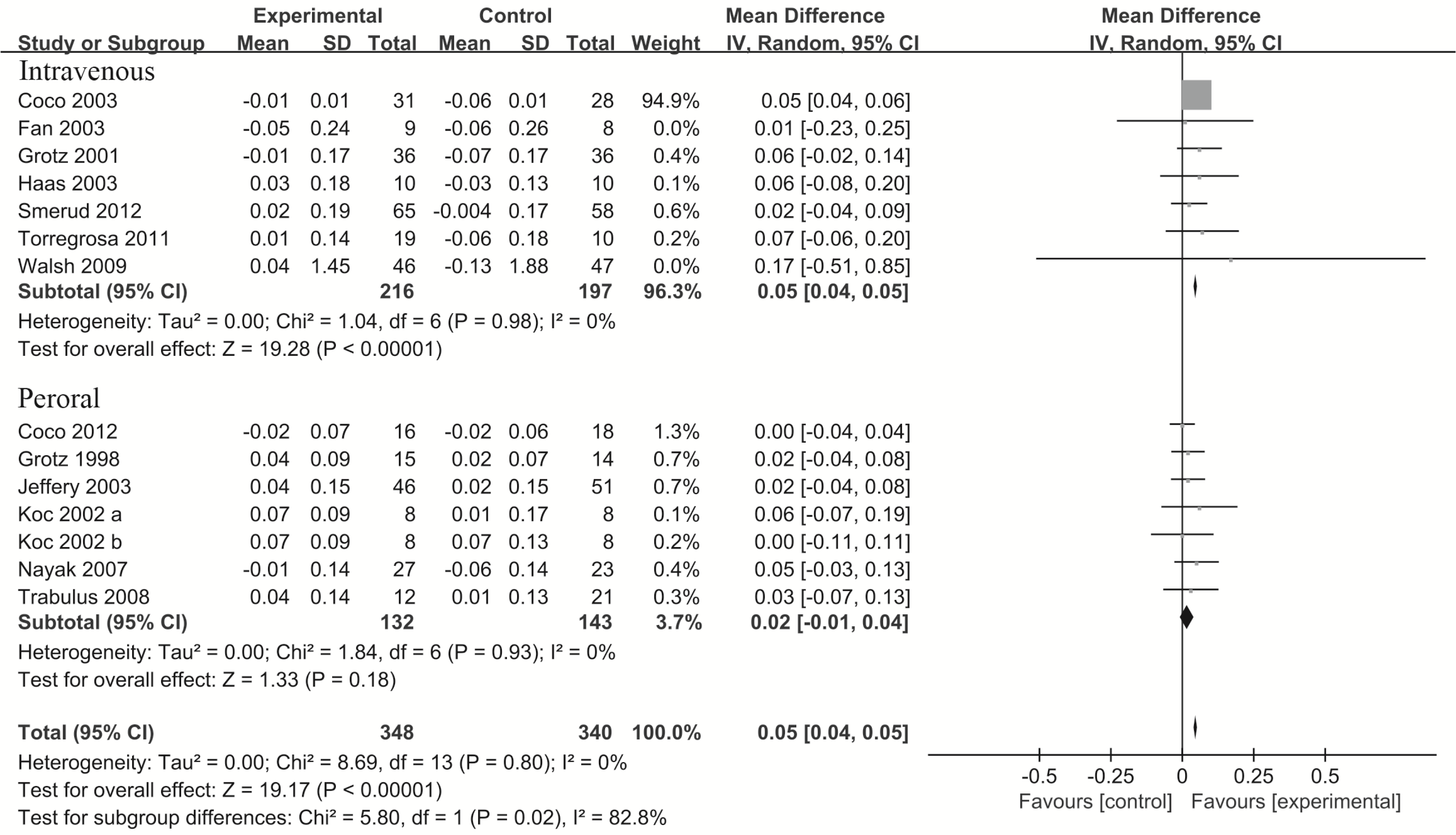


Supplemental Figure 4 Forest plot of absolute change in BMD at lumbar spine by subgroup analysis of study duration (short-term versus long-term): bisphosphonates vs. control


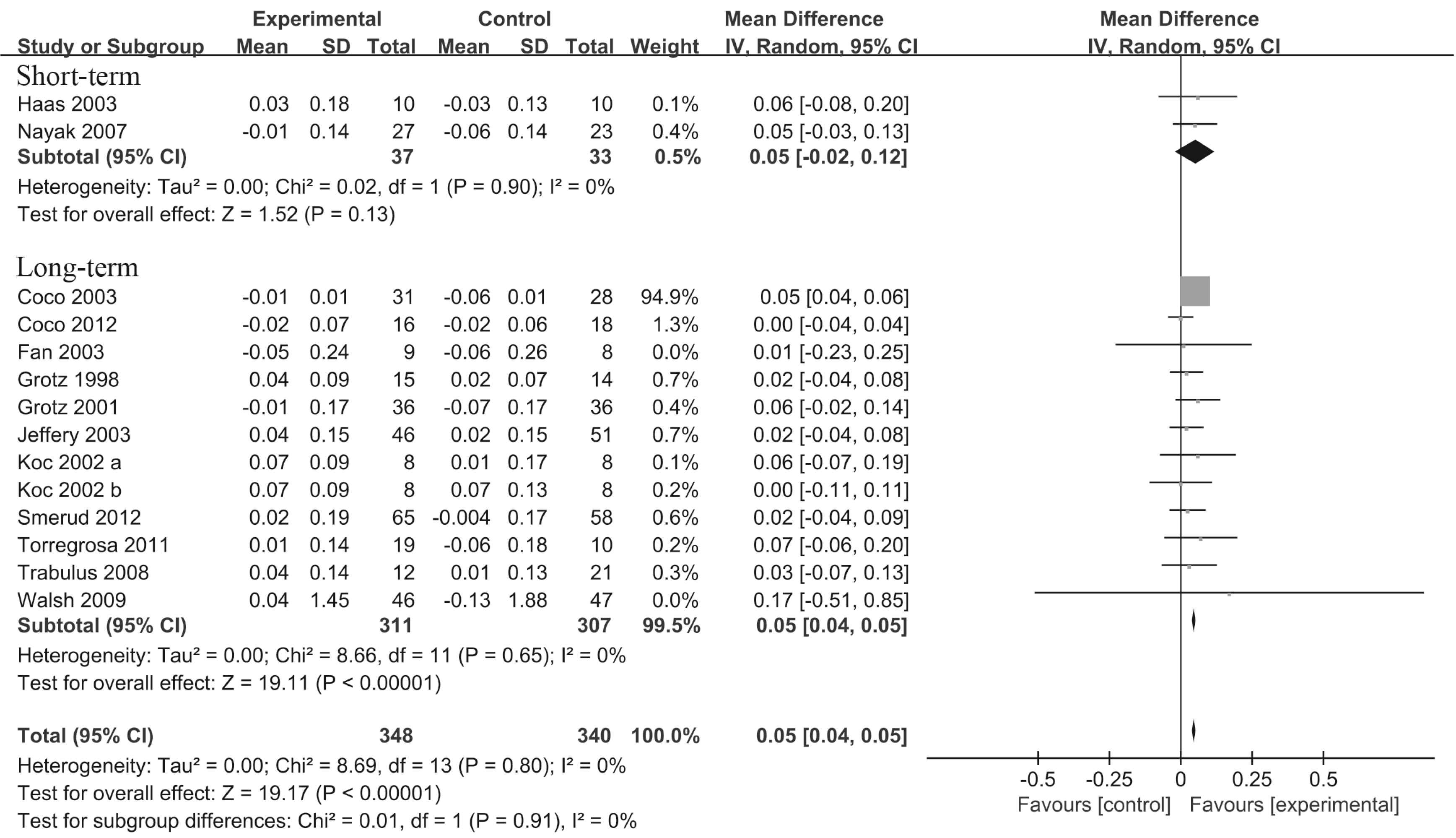


Supplemental Figure 5 Forest plot of absolute change in BMD at lumbar spine by subgroup analysis of bisphosphonates usage (continuous or intermittent): bisphosphonates vs. control


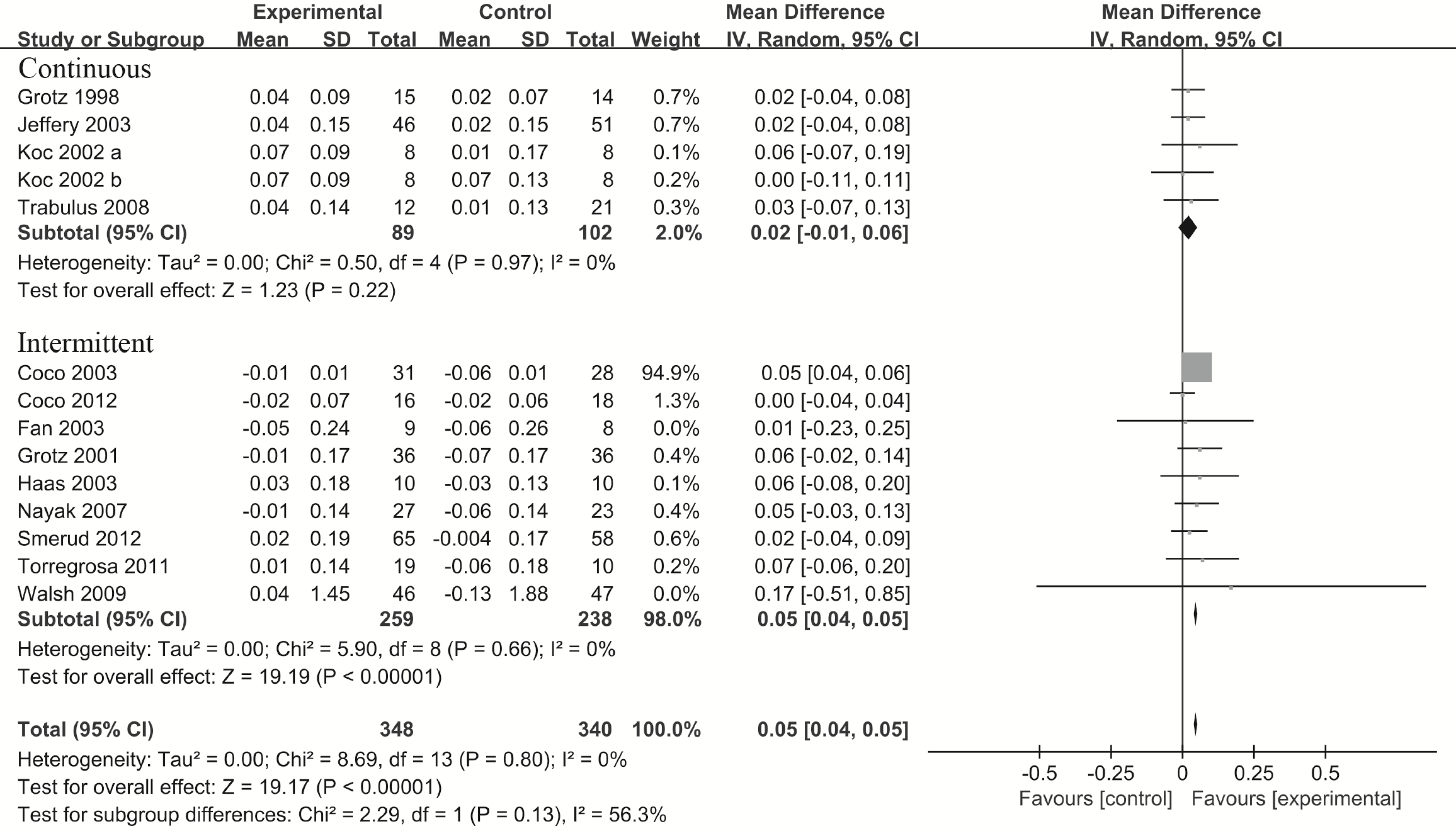


Supplemental Figure 6 Forest plot of absolute change in BMD at lumbar spine by subgroup analysis of treatment indication (prevention versus treatment of bone loss): bisphosphonates vs. control


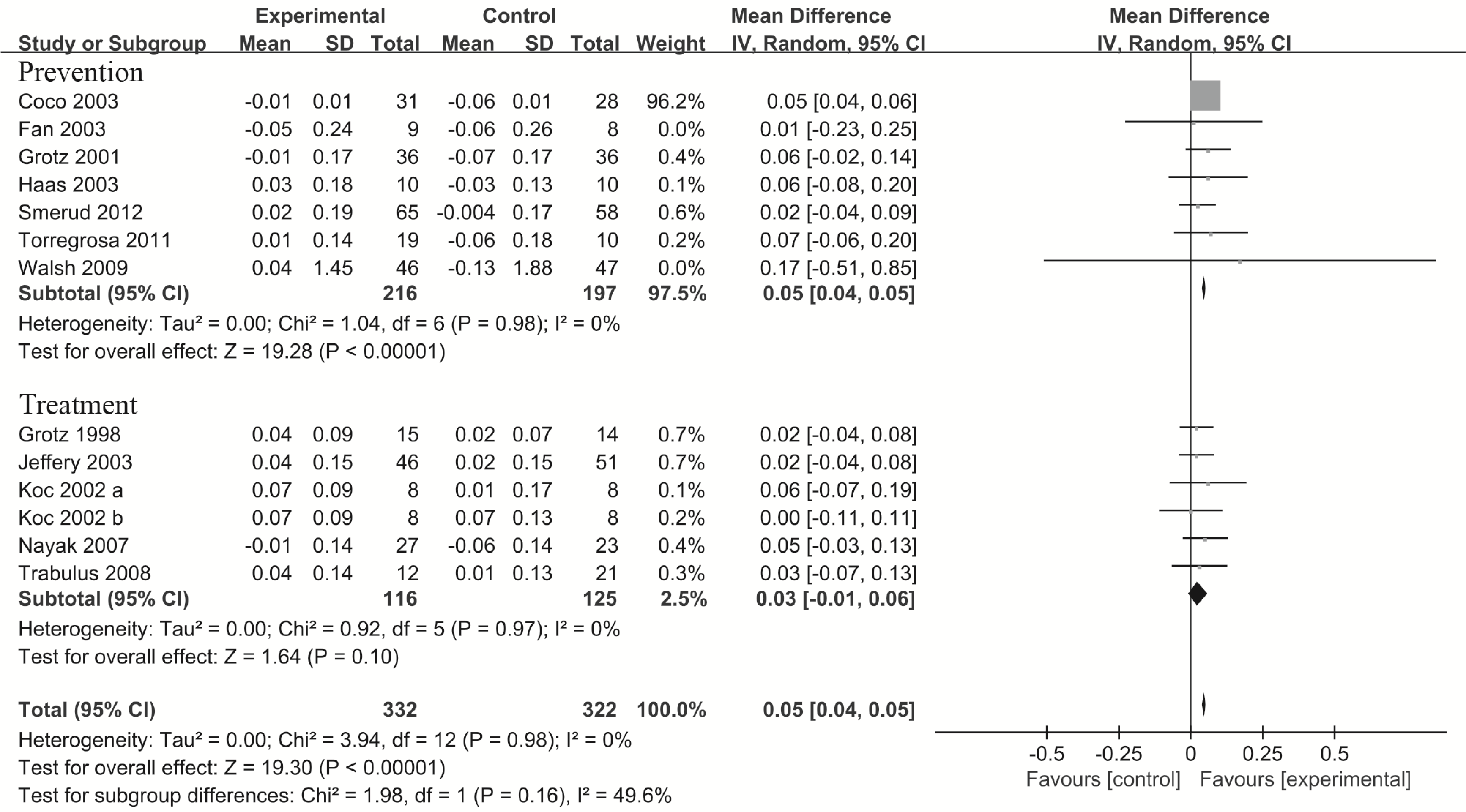


Supplemental Figure 7 Meta-regression analysis of influence of study duration on efficacy of bisphosphonates treatment

**
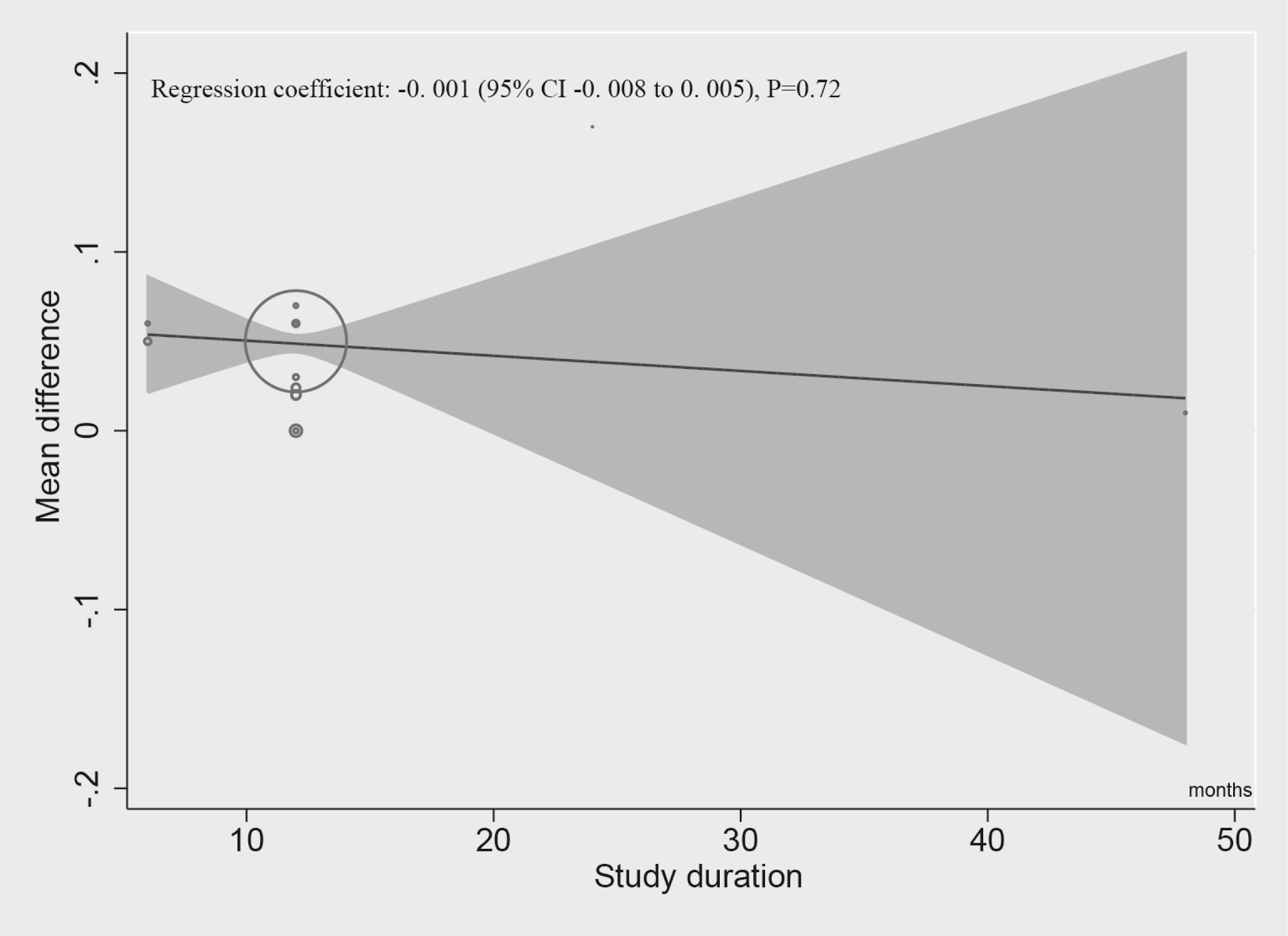
**
